# Metabolomic profiling for the identification of potential biomarkers involved in a laboratory azole resistance in *Candida albicans*

**DOI:** 10.1371/journal.pone.0192328

**Published:** 2018-02-02

**Authors:** Ling Li, ZeBin Liao, Yu Yang, Lei Lv, YingYing Cao, ZhenYu Zhu

**Affiliations:** 1 School of Pharmacy, Second Military Medical University, Shanghai, China; 2 Department of Radiation Medicine, Faculty of Naval Medicine, Second Military Medical University, Shanghai, China; 3 Department of Pharmacy, Shanghai Eastern Hepatobiliary Surgery Hospital, Shanghai, China; National University of Singapore, SINGAPORE

## Abstract

*Candida albicans*, one of the most common fungal pathogens, is responsible for several yeast infections in human hosts, being resistant to classically used antifungal drugs, such as azole drugs. Multifactorial and multistep alterations are involved in the azole resistance in *Candida albicans*. In this study, a FCZ-resistant *C*. *albicans* strain was obtained by serial cultures of a FCZ-susceptible *C*. *albicans* strain in incrementally increasing concentrations of FCZ. We performed an integrated profile of different classes of molecules related to azole resistance in *C*. *albicans* by combining several mass-spectrometry based methodologies. The comparative metabolomic study was performed with the sensitive and resistant strains of *C*.*albicans* to identify metabolites altered during the development of resistance to fluconazole, while the intervention strains and non-intervention strains of *C*.*albicans* to identify metabolites altered involved in cross-resistant to azole drugs. Our analysis of the different metabolites identified molecules mainly involved in metabolic processes such as amino acid metabolism, tricarboxylic acid cycle and phospholipid metabolism. We also compared the phospholipid composition of each group, revealing that the relative content of phospholipids significantly changed during the development of resistance to azole drugs. According with these results, we hypothesized that the metabolism shift might contribute to azole drugs resistance in *C*.*albicans* from multifactorial alterations. Our result paves the way to understand processes underlying the resistance to azole drugs in *C*. *albicans*, providing the basis for developing new antifungal drugs.

## Introduction

*Candida albicans* is an asexual opportunistic fungus that causes infections in immunocompromised and debilitated individuals[1]. Azole drugs are widely used in clinical practice having a good antibacterial power and low side effects[2]. Unfortunately, widespread uses of azole drugs have led to the rapid development of drug resistance which hampers the efficacy of current treatments for invasive mycoses. The considerable number of side effects mainly related to high-concentration use of drugs and prolonged therapies. The increase of drug resistance still make a serious clinical problem for fungal infections[3]. A strong mobilization of the scientific community allowed elucidation of the molecular mechanisms underlying fluconazole resistance in the yeasts *Candida*, of which a number of them could be extrapolated to other azole drugs, such as mutations in the *ERG11* gene, overexpression of efflux membrane transporters and overexpression of *ERG11*[4]. Metabolomics is a systems biology approach to study small molecules. It is a rapidly growing field that aims to profile as many low molecular weight metabolites as possible, rather than focus on single metabolites in cells, biofluids and tissue extracts[5, 6]. Almost all metabolomics approaches can be classified as targeted or untargeted. Targeted analyses always focus on a subset of known metabolites, while untargeted are global screening approaches[7]. Using bioinformatic and statistical tools, metabolomic profiles from different samples can be compared, identifying potential metabolite markers or patterns typical for a specific sample or condition. However, due to the wide range of metabolite concentrations and the diversity of their biochemical properties, no single analytical technique can provide a fully characterization of the metabolic profile of an organism. The best option is to use a combination of several analytical approaches. High-performance liquid chromatography mass spectrometry (HPLC-MS) and gas chromatography MS (GC-MS) are two widely used techniques for metabolomics analysis.

In this study, we designed to gain insight into alterations in metabolites associated with azole drugs resistance in laboratory *C*. *albicans* strains. Multiple metabolomic approaches were integrated for analyzing the metabolic fingerprinting and footprinting and profiling intracellular and extracellular metabolites associated with azole resistance in *C*. *albicans*. We adopted GC-MS and ultra-high performance liquid chromatography coupled with quadrupole time-of-flight MS (UHPLC-Q-TOF/MS) to obtain the metabolomic profiling. In addition, hydrophilic interaction liquid chromatography coupled with triple quadrupole MS (HILIC-QQQ/MS) method was used to profile phospholipid metabolism. Our results indicated that *C*. *albicans* responds to azole drugs by changing the expression of specific classes of metabolites. These metabolites were mainly related to anti-oxidative stress, and the function of cell membrane and mitochondrion. Although further studies are needed, our work intends to improve knowledge on the complex molecular networks involved in *C*. *albicans* resistance to azole drugs.

## Materials and methods

### Antifungal agents and chemicals

Fluconazole (FCZ), miconazole (MCZ), ketoconazole (KCZ) and the derivatizing reagent trimethylchlorosilane (TMCS), methoxyamine hydrochloride, pyridine and N-methyl-N-[trimethylsilyl]-trifluoroacetamide (MSTFA) were from Sigma Aldrich (St. Louis, MO, USA). Internal standards (IS) α-aminobutyric acid was from Dibo chemical and technology Co., Ltd (Shanghai, China). Ultrapure water was prepared with the Milli-Q water purification system (Millipore, Bedford, MA, USA) and solvents used for chromatographies were of HPLC-MS grade (Merck, Darmstadt, Germany). Other reagents were of analytical grade.

### Induction of resistant strains

*C*. *albicans* strain SC5314 was kindly provided by Professor William A. Fonzi. A single colony of the strain SC5314 was first inoculated into 10 mL of YPD medium (1% w/v yeast extract, 2% w/v peptone, and 2% w/v dextrose) and cells were grown at 30°C with constant agitation (200 r/min). Every day, an aliquot of the overnight culture containing 10^6^ cells was transferred into 10 mL of fresh medium containing twice their most recently measured minimal inhibitory concentration (MIC) of FCZ, and the cells were incubated overnight at 30°C with constant agitation. When the cultures reached a density of 10^8^ cells/mL, anothor aliquot containing 10^6^ cells was transferred into fresh medium and incubated as described above. Sensitive strains were grown without drug, while resistant strains were grown in twice their most recently measured MIC of fluconazole until they were grown steadily in 64 μg/mL of fluconazole. In order to study the developed cross-resistance to azole drugs, ketoconazole (KCZ) was set as an intervention drug. We divided these strains into 4 groups: sensitive strains (S group), resistant strains (R group), sensitive strains treated with ketoconazole (S+KCZ group), resistant strains treated with ketoconazole (R+KCZ group).

### Determination of MICs

During the experiment, MIC of fluconazole, miconazole and ketoconazole were determined at each sampling time by the broth microdilution method using 96 well plates according to the National Committee for Clinical Laboratory Standards (M27-A2) protocol[8]. The lowest concentration of the extract that produced no visible growth (no turbidity) after 24h when compared with the control tubes was considered as initial MIC of this generation.

### Culture conditions and determination of ketoconazole IC_50_

For the determination of the half maximal inhibitory concentration (IC_50_) of KCZ, *C*. *albicans* cells were suspended in YPD medium until reaching the optical density at 600 nm (OD600) of 0.1 and then allowed to reach the OD600 of 0.2 at 30°C. Several concentrations of KCZ were added and cells were incubated for 4 h at the same condition. Compared with control group, IC_50_ was 16 μg/mL and 32 μg/mL for sensitive and resistant strains, respectively. We thus set the concentration of KCZ for our experiments at 16 μg/mL.

### Sample preparation for UHPLC-Q-TOF/MS experiments

Intracellular metabolites of *C*. *albicans* SC5314 were isolated as previously described with slight modifications[9, 10]. Briefly, following the cultivation, all samples were rapidly washed with ice-cold ultrapure sterile water. For quenching, samples were resuspended in 1 mL of ice-cold methanol:water (60:40) at -80°C for 5 min and centrifuged at 5,000 ×g for 5 min. For the extraction, pellets were resuspended in 1 mL of boiling water (containing 200 μg/mL IS), for 15 min. Samples were then subject to three repeated freeze-thaw cycles (15 min in a -80°C refrigerator and at 60°C in hot water, conducted alternatively). After centrifugation at 10,000 ×g for 5 min at 4°C, supernatants were collected, transferred into a filter cap and centrifuged again at the same condition. Supernatants were collected and dissolved in the initial mobile phase (water: acetonitrile = 95:5) after freeze-drying. The cells were dried at room temperature to constant weight. The dry fungus weight was measured at least three times, and the mean value was considered as the biomass of each sample.

Extracellular metabolites were obtained by the centrifugation of culture medium at 5,000 ×g for 5 min. The supernatant was collected and dissolved in the initial mobile phase after being freeze-dried.

### Sample preparation for GC-MS experiments

For GC-MS analysis, intracellular metabolites were extracted as described above for UPLC-Q-TOF/MS, and derivatized after freeze-drying as follows: 75 μL of methoxyamine hydrochloride in pyridine (20 mg/mL) was added as first derivatization agent. The mixture was incubated at 70°C for 60 min. And 75 μL of MSTFA with 1% TMCS was added and incubated at 50°C for 60 min. Samples were then centrifuged at 10,000 ×g for 3 min at 4°C, supernatants were collected and mixed with 100 μL of heptane. The resulting solution (1 μL) was injected into the GC-MS system.

### Sample preparation for HILIC-QQQ/MS experiments

For the HILIC-QQQ/MS analysis, phospholipids were extracted according to the methyl tert-butyl ether (MTBE) method[11]. Briefly, each sample was resuspended in 1.5 mL of methanol and vortexed for 5 min. Then, 5 mL of MTBE was added, vortexed for other 30 min, and 1.25 mL of water was added. Samples were incubated for 10 min and centrifuged at 5,000 ×g for 5 min. The lower phase was extracted twice with 2 mL of solvent (MTBE: methanol: water = 10:3:2.5). Obtained samples were pooled and dried with N_2_.

### UHPLC-Q-TOF/MS analysis

The UHPLC-Q-TOF/MS analysis was performed on an Agilent 1290 Infinity LC system equipped with Agilent 6530 Accurate Mass Quadrupole Time-of-Flight mass spectrometer (Agilent, USA). An ACQUITY UPLC HSS T3 column (2.1 mm × 100 mm, 1.8 μm) keep at 40°C was employed for the peak separation. The mobile phase was a mixture of water with 0.1% formic acid (A) and acetonitrile (B) at a flow rate of 0.35 mL/min. The gradient elution was as follows: 5% B to 95% B for 15 min. The post time was set to 5 min for equilibrating the system and the injection volume was 4 μL. An electrospray ionization source (ESI) was used both in positive and negative mode. The optimized conditions were as follows: capillary voltage was 3.5 kV for both positive and negative mode; drying gas flow 11 L/min, gas temperature 350°C; nebulizer pressure 45 psi, fragmentor voltage 120 V, skimmer voltage 60 V. Data were collected in profile mode from *m/z* 50 to 1100. MS/MS analysis was carried out to study metabolite structures and the applied collision energy was from 10 to 30 eV.

### GC-MS analysis

The GC-MS analysis was performed with a Thermo Finnigan Trace GC Ultra instrument equipped with a DSQ II single quadrupole MS (Thermo Fisher, City, USA). A DB-5ms capillary column (30 m × 0.25 mm, 0.25 μm film thickness, Agilent, Santa Clara, CA, USA) was used for separation, and helium was used as carrier gas at a constant flow rate of 1.0 mL/min. The temperature of injection and interface was set to 260°C and 280°C, respectively. The GC oven temperature was initially set to 70°C for 3 min. Upon injection, the temperature was firstly increased to 220°C at a rate of 4°C/min and then to 310°C at a rate of 8°C/min, holding for 10 min. The temperature of the ion source was maintained at 250°C for electron ionization (EI). The full scan mode with a mass range of *m/z* 50–600 was used.

### HILIC-QQQ/MS analysis

Agilent 1290 Infinity LC system and 6460 triple quadrupole mass system equipped with HILIC Xbridge (3.0mm×100mm, 3.5 μm) keep at 3°C was employed to analyze phospholipid metabolites. The mobile phase was a mixture of water with 10 mM ammonium acetate (A) and 95% acetonitrile with 10 mM ammonium acetate (B) at a flow rate of 0.3 mL/min. The gradient elution was as follows: 100% B to 95% B for 2 min; 95% B for 7.5 min, changed to 75% B for 13 min. The post time was set to 2 min for equilibrating the system and the injection volume was 5 μL. An electrospray ionization source (ESI) was used both in positive and negative modes. The capillary voltage was 2500 V, drying gas flow 10 L/min, gas temperature 325°C and nebulizer pressure 50 psi. Other mass scan parameters are shown in Table 1.

**Table 1:** HILIC-QQQ/MS scan parameters.

Start Time (min)	Ionization mode	Scan Type	Mass range (m/z)	Fragmentor voltage(V)	Collision energy(eV)
0	-	Pre 152.9	600–1000	200	20
5.0	-	Pre 152.9	300–600	180	20
6.5	+	NL 141	600–1000	200	10
9.0	+	Pre 184.1	600–1000	180	20
11.5	+	NL 141	300–600	120	5
12.5	+	Pre 184.1	300–600	150	20

Pre: precursor-ion scanning; NL: neutral loss scanning

### Data processing

The raw data from GC-MS and UHPLC-Q-TOF/MS were converted to common data format (.cdf and.mzData). In the R software environment, the XCMS program was used to process MS data, with peak identification, retention time correction, automatic integration pretreatment, alignment and annotation. Metabolites not present in 80% of the samples were filtered. The raw data of extracellular and intracellular metabolites from LC-Q-TOF/MS are reported in supplementary materials (S1 and S2 Tables, respectively). GC-MS data inherently contain apparent variability and complexity, an untargeted filtration of ion peaks was used. Indeed, the most abundant fragment ion with the respective retention time (the time bin is 0.01 min) was kept while other ions were excluded. By applying this untargeted filtration, the data set was simplified and 174 ion peaks were obtained (supplementary materials S3 Table). HILIC-QQQ/MS data were processed by using the Agilent software pack. Then, according to the *m/z* and characteristic fragment ions, we inferred the number of carbon atoms and the number of double bonds in the fatty acid chain by using the network database (supplementary materials S4 Table). All the output data were normalized using the internal standard (IS) and dry weight of each sample before being exported to perform multivariate data analysis.

The identification of metabolites from the GC-MS was performed by combining mass spectra and database consultation (NIST11). For LC-MS data, the measured accurate mass and isotopic pattern were matched with database such as Metlin (http://metlin.scripps.edu/), YMDB (http://www.ymdb.ca/) and Lipid Maps (www.lipidmaps.org). MS/MS analysis was carried out to elucidate the metabolite structure and further validation was achieved by using literature data and reference standards.

### Statistical analysis

All the obtained data were analyzed by multivariate statistical data analysis. More in detail, the principal components analysis (PCA) and partial least-squares discriminant analysis (PLS-DA) were performed with SIMCA-P version 11.0 (Umetrics, Umea, Sweden)[12]. Unsupervised PCA was used to observe the separating trends and metabolic trajectories between each set of samples while PLS-DA was used to identify a metabolite candidate that could distinguish between different groups. Variable importance plot (VIP) was used to select interesting metabolite candidates with the threshold value of 1.0 (GC-MS) or 1.5 (UHPLC-Q-TOF/MS). The statistical significance of mean values was assessed by using the one-way ANOVA and the Tukey’s post hoc test by SPSS 17.0. Results were considered significant when p < 0.05.

## Results

### The sensitivity of the strain to azole drugs

**Fig 1:**
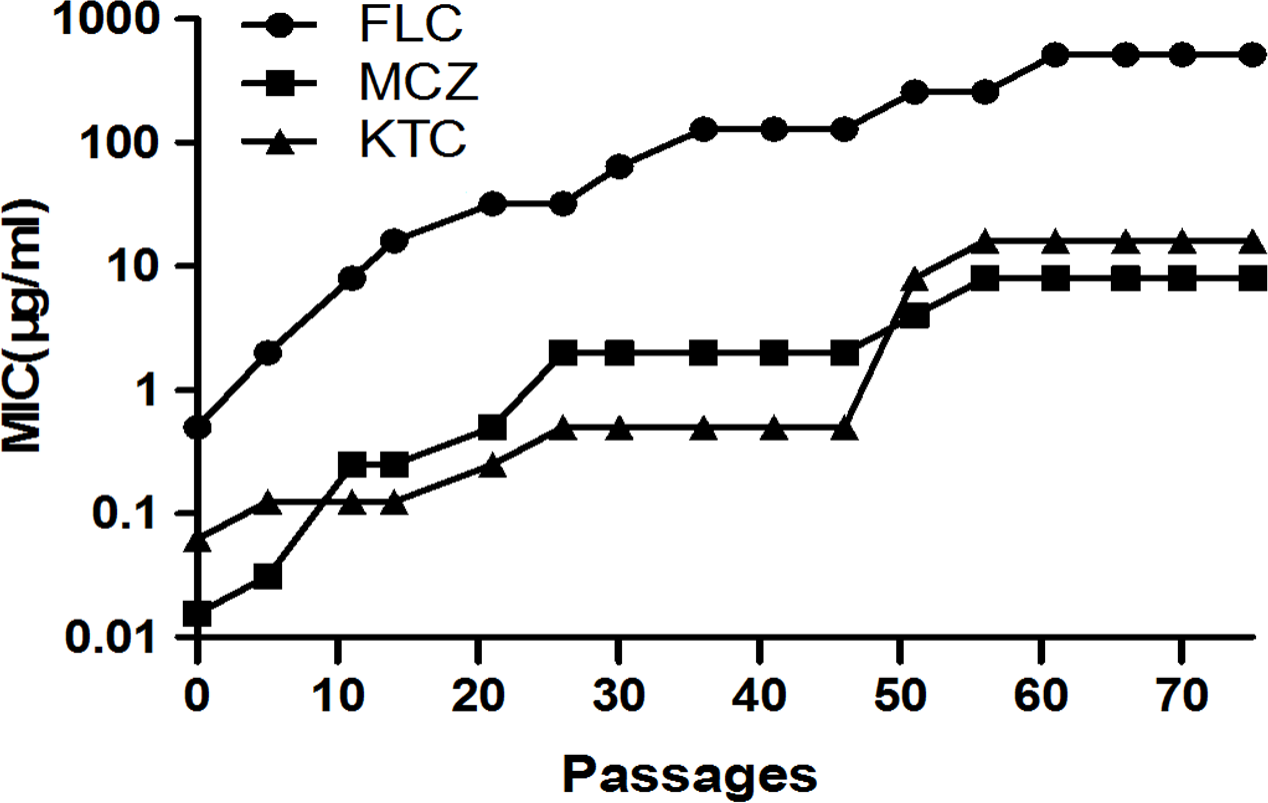
Variations of MIC values of FCZ, MCZ and KCZ for the *C*. *albicans* strain SC5314 grown in medium containing twice their most recently measured MIC of FCZ. The result showed that the increase in fluconazole MIC was accompanied by a corresponding increase in resistance to miconazole and ketoconazole.

To obtain the resistant *C*. *albicans* strain in the laboratory, *C*. *albicans* SC5314 cells (FCZ MIC = 0.5 μg/mL) were cultivated in YPD medium with twice their most recently measured MIC of fluconazole. Reduced sensitivity to FCZ was detected after 5 passages, the high-level resistant strain (FCZ MIC = 256 μg/mL) was generated after about 51 passages and the next generations were cultivated in YPD medium with fluconazole (MIC = 512 μg/mL) and retained the resistant phenotype for more than 15 passages (Fig 1). The sensitivity testing showed that the increase in fluconazole MIC was accompanied by a corresponding increase in resistance to miconazole and ketoconazole (Fig 1). All the strains exposed to fluconazole adapted to the presence of drug, showing increased azole cross-resistance.

### Metabolic profiling analysis

**Fig 2:**
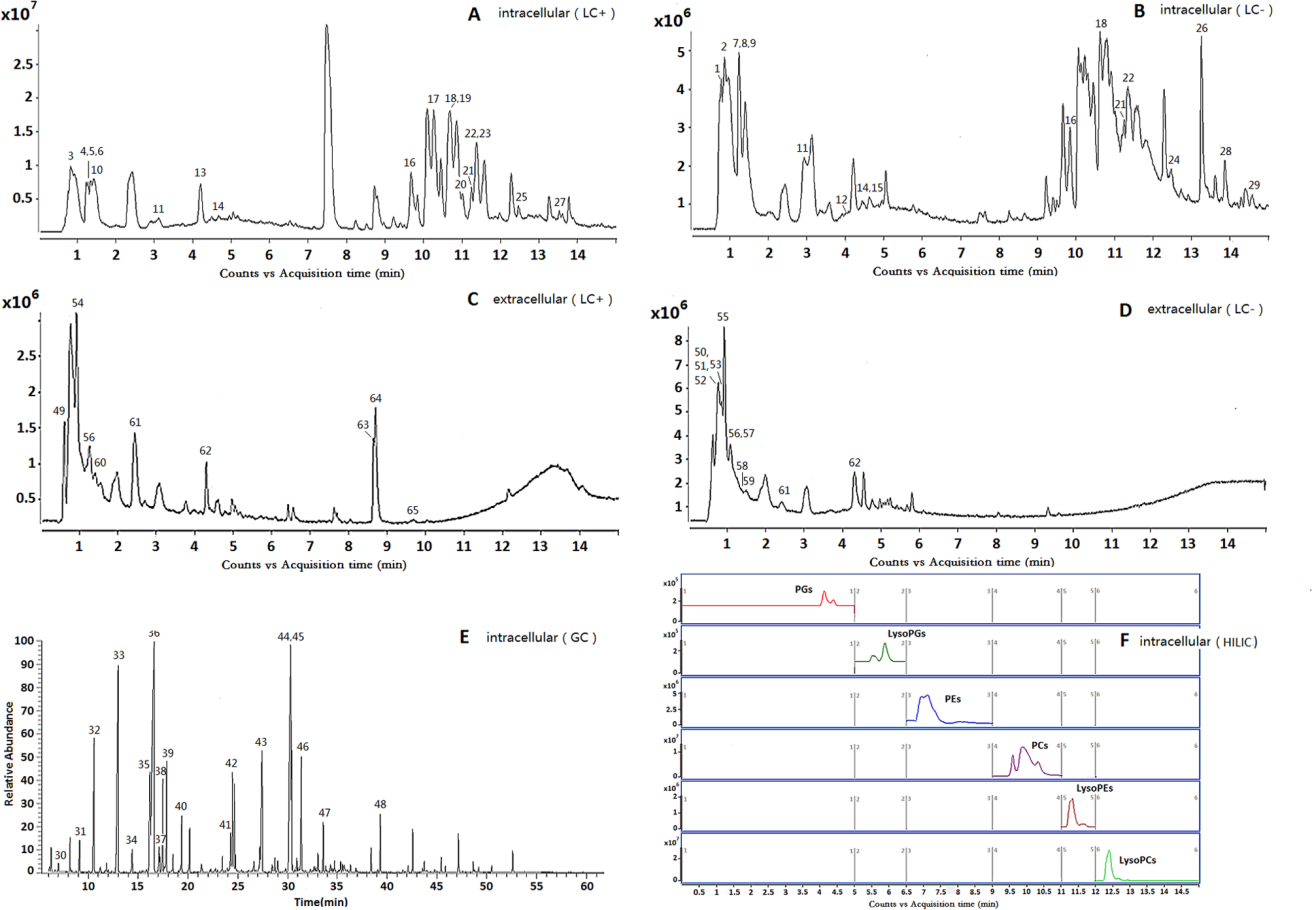
The typical total ions current chromatograms. (A&B)Intracellular sample separated on UHPLC-Q-TOF/MS in positive and negative ionization mode; (C&D)Extracellular sample separated on UHPLC-Q-TOF/MS in positive and negative ionization mode; (E)Intracellular sample separated on GC-MS; (F)Extracted ion chromatography on HILIC-QQQ/MS.

**Fig 3:**
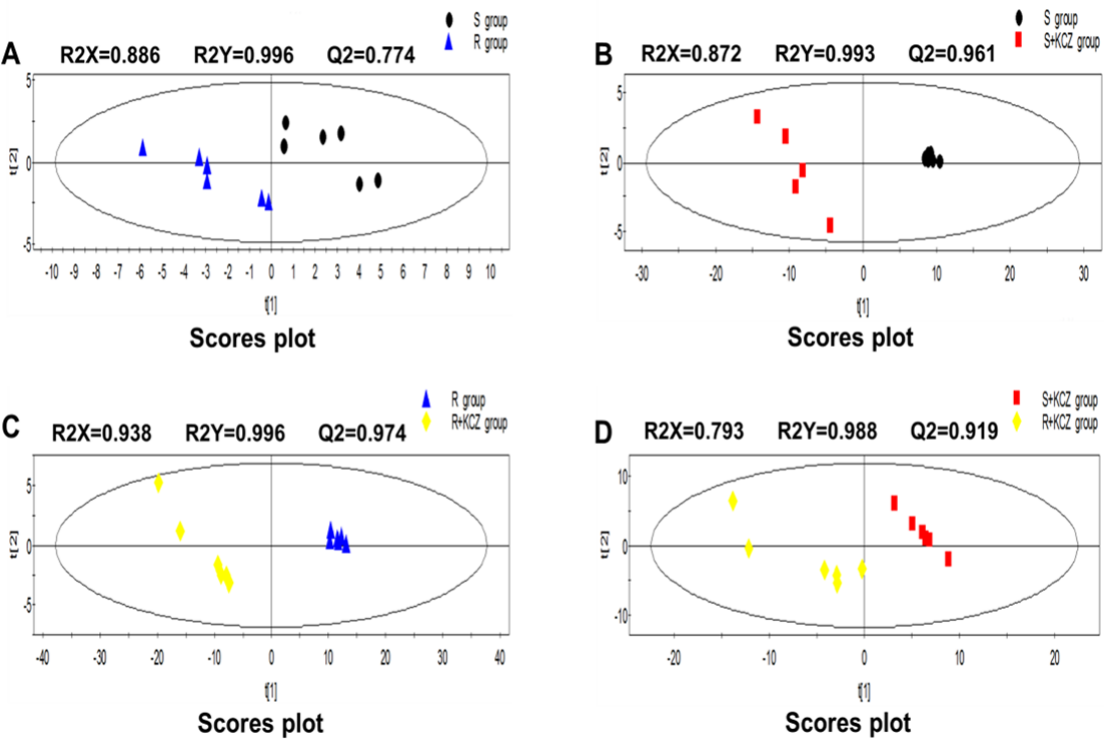
PLS-DA scores plot of intracellular metabolites from GC-MS. (A)S group vs R group; (B)S group vs S+KCZ group; (C)R group vs R+KCZ group; (D)S+KCZ group vs R+KCZ group.

**Fig 4:**
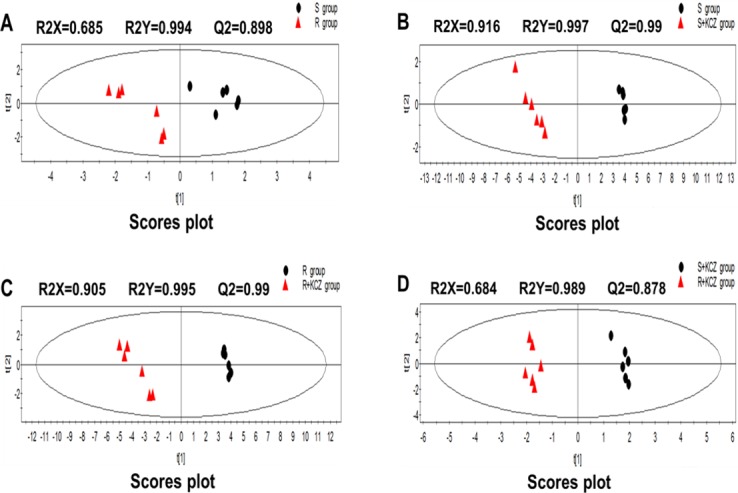
PLS-DA scores plot of intracellular metabolites from LC-Q-TOF/MS. (A)S group vs R group; (B)S group vs S+KCZ group; (C)R group vs R+KCZ group; (D)S+KCZ group vs R+KCZ group.

**Fig 5:**
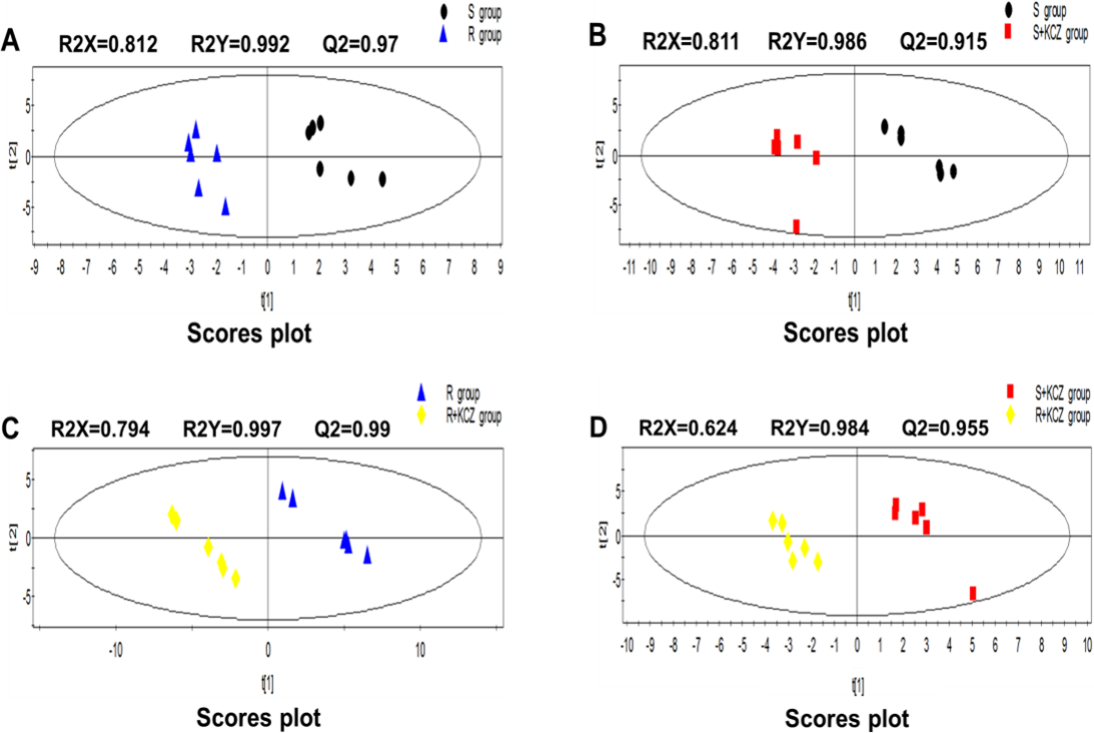
PLS-DA scores plot of extracellular metabolites from LC-Q-TOF/MS. (A)S group vs R group; (B)S group vs S+KCZ group; (C)R group vs R+KCZ group; (D)S+KCZ group vs R+KCZ group.

In this study, changes in intracellular and extracellular metabolites associate with azole resistance of *C*. *albicans* were evaluated. The typical total ion current chromatograms (TICs) are shown in Fig 2A–2E. In order to detect expression changes in metabolites due to external stimuli (i.e. KCZ treatment) or between strains themselves, four models were build for comparing: S group *vs* R group; S group *vs* S+KCZ group; R group *vs* R+KCZ group; S+KCZ group *vs* R+KCZ group (Fig 3, Fig 4, Fig 5). After merging redundant variables from the same metabolite, several molecules were selected as statistically significant (p<0.05; Table 2). According to this algorithm, we identified 48 and 17 differential molecules related to resistance among intracellular and extracellular metabolites, respectively. Moreover, by using the double factor analysis of variance, 25 significantly differential variables were identified as interacting (we set the *C*. *albicans* strain as resistant or not as factor A, and the presence of drug treatment or not as factor B, Table 3).

**Table 2:** The number of differential metabolites from each model.

Models	UHPLC-Q-TOF/MS		GC-MS
	intracellular extracellular		intracellular
S group vs R group	8	9	8
S group vs S+KCZ group	19	7	17
R group vs R+KCZ group	21	9	15
S+KCZ group vs R+KCZ group	8	6	6
Interaction	15	2	8

**Table 3:** Related metabolites and their metabolic pathway.

No.	Mass	RT	Column	Formula	Metabolite	Related pathway	S vs R^a^		S vs S+KCZ^b^		R vs R+KCZ^c^		S+KCZ vs R+KCZ^d^	
							Trend	FC	Trend	FC	Trend	FC	Trend	FC
**intracellular**														
1	155.0695	0.74	LC	C_6_H_9_N_3_O_2_	Histidine^e^	Amino acid metabolism	——		↓***	0.4	↓***	0.5	——	
2	117.0426	0.89	LC	C_4_H_7_NO_3_	L-Aspartate-4-semi aldehyde^f^^g^	Amino acid metabolism	↑***	2.0	——		——		——	
3	257.1028	0.96	LC	C_8_H_20_NO_6_P	Glycerophosphocholine^e^^g^	Phospholipid metabolism	↑**	2.0	↑***	4.3	↑***	3.9	↑***	1.8
4	149.0510	1.24	LC	C_5_H_11_NO_2_S	Methionine^e^^g^	Amino acid metabolism	——		↓***	0.4	↓***	0.2	——	
5	307.0838	1.24	LC	C_10_H_17_N_3_O_6_S	Glutathione^e^^g^	Glutathione metabolism	——		——		↑***	2.8	↑***	2.2
6	136.0385	1.24	LC	C_5_H_4_N_4_O	Hypoxanthine^e^^g^	Purine metabolism	——		——		——		↑***	2.4
7	192.0270	1.25	LC	C_6_H_8_O_7_	Citrate^e^	Citrate cycle	——		↓***	0.1	↓***	0.2	↑***	2.7
8	146.0215	1.25	LC	C_5_H_6_O_5_	2-Oxoglutarate^e^	Citrate cycle	↑**	1.6	↑***	3.0	↑***	2.0	——	
9	244.0695	1.25	LC	C_9_H_12_N_2_O_6_	Uridine^e^	Pyrimidine metabolism	——		——		↓***	0.2	——	
10	137.0477	1.66	LC	C_7_H_7_NO_2_	N-Methyl nicotinate^f^^g^	Nicotinate metabolism	↓***	0.2	——		——		——	
11	219.1107	3.01	LC	C_9_H_17_NO_5_	Pantothenic acid^e^^g^	Vitamin metabolism	——		↑***	3.2	↑***	2.0	——	
12	177.0460	4.02	LC	C_6_H_11_NO_3_S	N-Formylmethionine^f^^g^	Amino acid metabolism	——		↓***	0.2	↓***	0.2	——	
13	135.0545	4.19	LC	C_5_H_5_N_5_	Adenine^e^	Purine metabolism	↑**	1.7	——		——		↑**	2.1
14	224.0797	4.61	LC	C_10_H_12_N_2_O_4_	3-Hydroxy-kynu renine^f^^g^	Tryptophan metabolism	——		——		↑***	5.5	↑***	3.7
15	180.0899	4.62	LC	C_9_H_12_N_2_O_2_	5-Hydroxy-kynu renamine^f^^g^	Tryptophan metabolism	——		——		——		↑***	2.0
16	397.2593	9.85	LC	C_18_H_40_NO_6_P	Phytosphingosine-1-P^e^	Sphingolipid metabolism	↑***	1.5	——		——		↑*	1.5
17	493.3168	10.36	LC	C_24_H_48_NO_7_P	LysoPC(16:1)^e^	Phospholipid metabolism	↑**	1.7	↑**	2.6	↑***	2.1	——	
18	477.2855	10.71	LC	C_23_H_44_NO_7_P	LysoPE(18:2)^f^	Phospholipid metabolism	——		↑***	3.6	↑***	2.8	——	
19	519.3325	10.78	LC	C_26_H_50_NO_7_P	LysoPC(18:2)^f^	Phospholipid metabolism	——		↑***	2.5	↑***	2.0	——	
20	495.3325	11.03	LC	C_24_H_50_NO_7_P	LysoPC(16:0)^e^^g^	Phospholipid metabolism	——		↑***	7.8	↑***	4.5	——	
21	453.2855	11.20	LC	C_21_H_44_NO_7_P	LysoPE(16:0)^e^^g^	Phospholipid metabolism	——		↑***	10.2	↑***	8.3	——	
22	479.3012	11.43	LC	C_23_H_46_NO_7_P	LysoPE(18:1)^f^	Phospholipid metabolism	——		↑***	3.5	↑***	3.4	——	
23	521.3481	11.49	LC	C_26_H_52_NO_7_P	LysoPC(18:1)^f^	Phospholipid metabolism	——		↑***	2.6	↑***	1.9	——	
24	481.3168	12.42	LC	C_23_H_48_NO_7_P	LysoPE(18:0)^e^^g^	Phospholipid metabolism	——		↑***	17.1	——		——	
25	523.3638	12.48	LC	C_26_H_54_NO_7_P	LysoPC(18:0)^e^	Phospholipid metabolism	——		↑***	4.3	↑***	4.1	——	
26	278.2246	13.28	LC	C_18_H_30_O_2_	γ-Linoleic acid^f^^g^	Fatty acid metabolism	——		↑***	9.6	↑***	6.2	——	
27	511.4964	13.50	LC	C_32_H_65_NO_3_	Cer(d18:0/14:0)^f^	Sphingolipid metabolism	↑*	1.9	——		——		——	
28	280.2402	13.88	LC	C_18_H_32_O_2_	Linoleic acid^e^^g^	Fatty acid metabolism	——		↑***	4.0	↑***	3.5	——	
29	282.2559	14.58	LC	C_18_H_34_O_2_	Oleate^e^	Fatty acid metabolism	——		↑***	4.3	↑***	4.3	——	
30	103.0633	7.08	GC	C_4_H_9_NO_2_	N,N-Dimethyl glycine	Amino acid metabolism	↓*	0.7	↑***	4.4	↑**	5.6	——	
31	90.0317	9.23	GC	C_3_H_6_O_3_	Lactate	Citrate cycle	↑*	1.4	——		——		↓**	0.6
32	90.0550	10.63	GC	C_3_H_7_NO_2_	Alanine	Amino acid metabolism	——		↑*	2.4	↑***	3.3	——	
33	131.0946	12.43	GC	C_6_H_13_NO_2_	Leucine	Amino acid metabolism	——		↑***	13.2	——		——	
34	117.0790	14.43	GC	C_5_H_11_NO_2_	Valine	Amino acid metabolism	——		↑***	6.9	↑***	7.2	——	
35	97.9769	16.50	GC	H_3_PO_4_	Phosphate^g^	Energy metabolism	——		↑**	2.9	——		——	
36	92.0473	16.57	GC	C_3_H_8_O_3_	Glycerol^g^	Stress response	↓*	0.7	↑***	3.2	↑***	4.7	↑*	1.4
37	115.0633	17.20	GC	C_5_H_9_NO_2_	Proline	Amino acid metabolism	↓*	0.8	↑***	4.6	↑***	1.8	——	
38	75.0320	17.51	GC	C_2_H_5_NO_2_	Glycine	Amino acid metabolism	——		↑**	1.5	↑***	1.5	——	
39	90.0317	17.89	GC	C_3_H_6_O_3_	Succinate^g^	Citrate cycle	——		↑***	2.5	↑***	3.8	——	
40	105.0426	19.36	GC	C_3_H_7_NO_3_	Serine	Amino acid metabolism	↑*	1.4	↑**	3.8	——		——	
41	133.0375	24.43	GC	C_4_H_7_NO_4_	Aspartate	Amino acid metabolism	↓**	0.7	↑***	11.2	↑***	13.5	——	
42	103.0633	24.64	GC	C_4_H_9_NO_2_	γ-Aminobutryic acid^g^	Citrate cycle	——		↑***	23.5	↑***	47.7	↑***	2.1
43	146.0691	27.43	GC	C_5_H_10_N_2_O_3_	Glutamine^g^	Amino acid metabolism	↓***	0.6	↑***	1.9	↑***	3.9	↑*	1.3
44	152.0685	30.29	GC	C_5_H_12_O_5_	Arabitol	Stress response	——		↑***	3.5	↑***	5.5	——	
45	152.0685	30.29	GC	C_5_H_12_O_5_	Ribitol^g^	Pentose phosphate metabolism	↓**	0.6	↑***	3.2	↑***	5.5	——	
46	172.0137	31.35	GC	C_3_H_9_O_6_P	Glycerophosphate	Phospholipid metabolism	——		↑***	50.8	↑***	68.9	——	
47	188.1161	33.56	GC	C_8_H_16_N_2_O_3_	N-Acetyl-L-lysine^g^	Amino acid metabolism	——		↑***	8.2	↑***	18.1	↑*	1.5
48	180.0634	39.33	GC	C_6_H_12_O_6_	Inositol^g^	Inositol phosphate metabolism	——		——		——		↓*	0.7
**extracellular**														
49	202.2157	0.59	LC	C_10_H_26_N_4_	Spermine^e^	Spermine metabolism	↑**	1.4	↓***	0.8	——		↓*	0.7
50	152.0685	0.78	LC	C_5_H_12_O_5_	Ribitol^e^	Pentose phosphate metabolism	↓**	0.8	——		——		↓*	0.8
51	180.0634	0.78	LC	C_6_H_12_O_6_	Glucose^e^	Energy metabolism	——		↑**	1.1	——		——	
52	120.0436	0.79	LC	C_5_H_4_N_4_	Purine^e^	Purine metabolism	——		↓**	1.3	——		——	
53	268.0808	0.85	LC	C_10_H_12_N_4_O_5_	Inosine^e^	Purine metabolism	↑*	1.2	↑**	1.2	↑***	1.1	——	
54	117.0790	0.86	LC	C_5_H_11_NO_2 _	Valine^e^^g^	Amino acid metabolism	↓***	0.6	——		——		↓***	0.6
55	175.0481	0.95	LC	C_6_H_9_NO_5_	N-Acetyl-aspartic acid^f^	Amino acid metabolism	↓*	0.8	——		——		——	
56	192.0270	1.10	LC	C_6_H_8_O_7_	Citrate^e^	Citrate cycle	↑**	1.6	↓**	0.6	↓***	0.5	——	
57	134.0579	1.15	LC	C_5_H_10_O_4 _	2-Deoxy-Ribose^f^	Pentose phosphate metabolism	↑**	1.3	——			——	↑*	1.8
58	181.0739	1.27	LC	C_9_H_11_NO_3_	Tyrosine^e^	Amino acid metabolism	——		↑**	1.2	↑***	1.1	——	
59	152.0797	1.34	LC	C_4_H_12_N_2_O_4_	succinate^e^	Citrate cycle	↑***	1.3	——		——		↑***	1.2
60	131.0946	1.40	LC	C_6_H_13_NO_2_	Leucine^e^^g^	Amino acid metabolism	——		——		↓***	0.9	↓*	0.8
61	165.0790	2.44	LC	C_9_H_11_NO_2_	Phenylalanine^e^	Amino acid metabolism	——		——		↑*	1.2	——	
62	204.0899	4.29	LC	C_11_H_12_N_2_O_2_	Tryptophan^e^	Tryptophan metabolism	——		——		↑**	1.2	——	
63	317.2930	8.69	LC	C_18_H_39_NO_3_	Phytosphingosine^e^	Sphingolipid metabolism	——		↑*	1.9	——		——	
64	271.2511	8.91	LC	C_16_H_33_NO_2_	Sphingosine^f^	Sphingolipid metabolism	↑*	1.6	↑*	2.5	——		——	
65	301.2981	9.66	LC	C_18_H_39_NO_2_	Sphinganine^e^	Sphingolipid metabolism	——		↑**	2.7	——		——	

Abbreviations: RT, retention time; LysoPC, lysophosphatidylcholine; LysoPE, lysophosphatidylethanolamine; Cer, ceramide

^a^ The trend and fold change (FC) of relative amounts of the R group compared to the S group;(↑): up-regulated. (↓): down-regulated. (* p<0.05; ** p <0.01; ***p< 0.001).

^b^ The trend and fold change of relative amounts of the S+KCZ group compared to the S group

^c^ The trend and fold change of relative amounts of the R+KCZ group compared to the R group

^d^ The trend and fold change of relative amounts of the R+KCZ group compared to the S+KCZ group.

^e^ Metabolites validated with standard sample

^f^ Metabolites putatively annotated by library searching

^g^ Metabolites of the interaction.

### Differential metabolites identified among different strains

Partial least squares discriminant analysis (PLS-DA) were performed to screen the differences of metabolites between sensitive and resistant strains. According to the VIP value and one-way analysis of variance (ANOVA, p<0.05), 25 variables were selected as candidates of potential biomarkers between sensitive and resistant strains. These potential biomarkers were mapped onto 12 principal pathways by using the KEGG PATHWAY Database (http://www.genome.jp/kegg/). Compared with the sensitive strain of *C*. *albicans*, the levels of N-Methyl nicotinate, N, N-dimethyl glycine, Glycerol, proline, Ribitol, L-aspartate, glutamine, valine, and N-Acetyl-aspartic acid were significantly decreased in the resistant strains, while the other metabolites including L-Aspartate-4-semi aldehyde, Glycerophosphocholine, 2-Oxoglutarate, Adenine, Phytosphingosine-1-P, LysoPC(16:1), Cer(d18:0/14:0), Lactate, Serine, Spermine, Inosine, Citrate, 2-Deoxy-Ribose, succinate and sphingosine were increased.

### Differential metabolites associate with drug stress

Among the sensitive and resistant strains tretment with KCZ compared with no drug intervention, 41 differential metabolites were identified as potential biomarkers related to drug stress. Four of them (i.e. serine, sphingosine, dihydrosphingosine and phytosphingosine) were detected only in the sensitive strain, while 7 (i.e. glutathione, tryptophan, uridine, 3-hydroxy-kynurenine, phenylalanine, purine and glucose) only in the resistant strains. These compounds are mainly involved in processes such as amino acid metabolism, stress response, sphingolipid metabolism, and phospholipid metabolism. In our results, most of the amino acid levels (i.e. alanine aspartate, glutamine, glycine, N, N-dimethyl glycine, N-acetyl lysine, proline, valine, and tyrosine) were increased both in sensitive and resistant strains after the treatment with KCZ.

### Differential metabolites related to drug resistance level

Twenty significantly differential metabolites were identified as potential biomarkers related to drug resistance level. Compared with S+KCZ group, it was found that fourteen of 20 metabolites, including glycerophosphocholine, glutathione, hypoxanthine, citrate, adenine, 3-hydroxy-kynurenine, 5-hydroxy-kynurenamine, phytosphingosine-1-P, glycerol, γ-aminobutryic acid, glutamine, 2-deoxy-ribose, N-acetyl-L-lysine and succinate, were up-regulated in the R+KCZ group, while the other six metabolites including lactate, inositol, spermine, ribitol, valine and leucine were reduced. These differentially expressed compounds are mainly related to the antioxidant activity or the action of drugs. For example, *C*. *albicans* regulates the content of VB6 by adjusting valine metabolism in order to assess the antioxidant activity. Besides, regulation of glutathione, polyamine and 3-Hydroxy-kynurenine may reduce intracellular reactive oxygen species, thus reducing the fungal sensitivity to drugs.

### Phospholipid metabolism analysis

**Fig 6:**
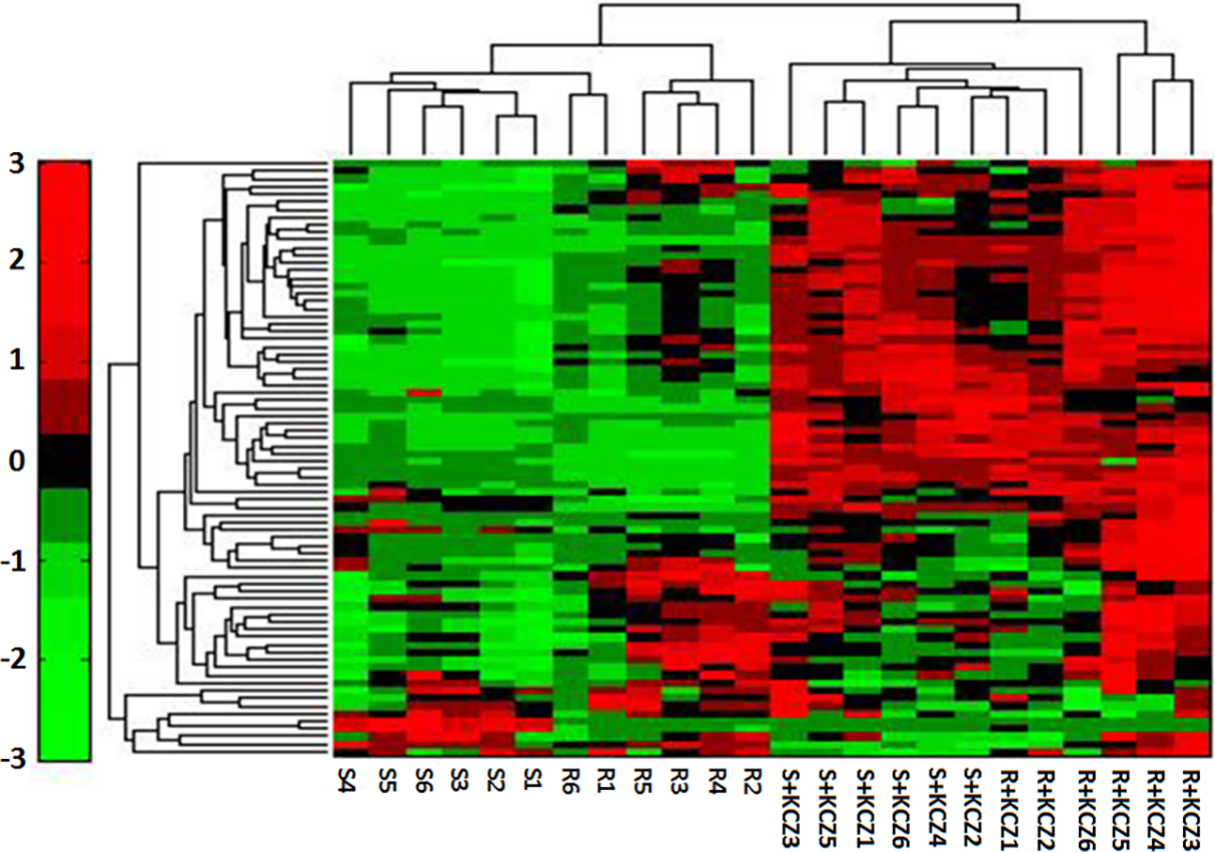
Heatmap of glycerophospholipid from HILIC-QQQ/MS. Colors represent fold change in the metabolite concentration in different groups. Red indicates increased concentration levels of metabolites; green indicates decreased concentration levels of metabolites.

Lipids, in addition to being structural and metabolic components of yeast cells, also appear to be responsible for drug resistance in *C*. *albicans*. Considering the central role of phospholipid metabolism in several biochemical processes, we decided to perform the phospholipid metabolomic analysis. 79 phospholipidic compounds were identified by HILIC-QQQ/MS (Fig 2F). All these metabolites are reported in a heatmap,including 28 phosphatidylcholines (PC), 10 lysophosphatidylcholines (LysoPC), 20 phosphatidyl ethanolamines (PE), 6 lysophosphatidyl ethanolamines (LysoPE), 12 phosphatidylglycerols (PG) and 3 lysophosphatidylglycerols (LysoPG) (Fig 6). We also compared the percentage of glycerol phospholipid contained in each group, observing that phosphatidylcholine was the most abundant compound. Finally, glycerol-phospholipid fatty acids analysis showed that resistant strains of *C*. *albicans* presented the highest content of glyceryl phosphatide fatty acids, which contain more than 34-C and an odd number of C atoms (Table 4, Table 5).

**Table 4:** The result of total number of carbons in the fatty acid chains of phosphoglyceride.

No. of carbons	S group		R group		S+KCZ group		R+KCZ group	
	mean	S.D.	mean	S.D.	mean	S.D.	mean	S.D.
<34-C	5.72	0.27	5.45	0.23	5.47	0.11	5.27	0.13
34-C	30.23	0.78	34.97	0.78	28.65	0.68	28.97	1.10
36-C	42.99	1.03	47.65	0.91	39.21	1.01	43.48	0.82
38-C	0.58	0.01	0.75	0.08	0.69	0.04	0.74	0.05

The data is represented as % of total PGLs mass spectral signal(n = 6)

**Table 5:** The result of odd chain fatty acid of phosphoglyceride.

No. of carbons	S group		R group		S+KCZ group		R+KCZ group	
	mean	S.D.	mean	S.D.	mean	S.D.	mean	S.D.
33-C	1.25	0.06	1.20	0.12	0.82	0.02	0.76	0.05
35-C	1.38	0.10	1.36	0.05	1.28	0.07	1.27	0.06

The data is represented as % of total PGLs mass spectral signal(n = 6)

## Discussion

To investigate biological mechanisms of azole resistance in *C*. *albicans*, a laboratory azole resistant *C*. *albicans* strain was obtained by serial cultures of a FCZ susceptible *C*. *albicans* strain in inhibitory concentrations of FCZ. This resistant strain possessed high-level and stable resistant characteristic, as well as cross-resistance to two other azole antifungal agents. Comparative analysis of metabolomics in groups showed that the differentially expressed metabolites were found to be involved in multiple biochemical functions. It is reasonable to assume that many of the observed alternations are somewhat related to azole resistance in *C*. *albicans*.

**Fig 7:**
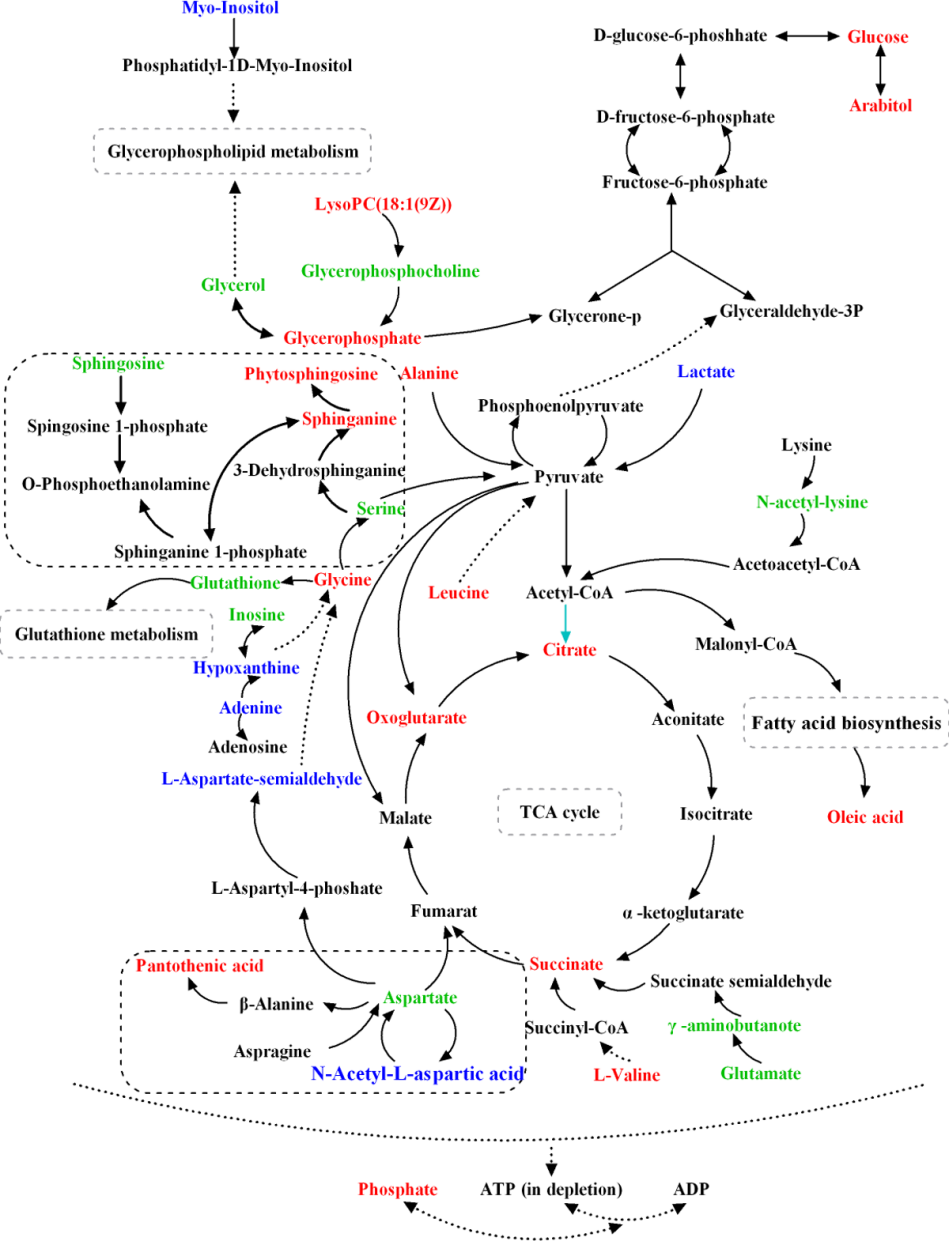
Schematic overview of the main differences in the metabolite change associated with drug resistance of *Candida albicans*. The differentially produced metabolites are mainly involved in amino metabolism, sphingolipid metabolism, TCA cycle, oxidative stress, glutathione metabolism and lipid metabolism. Metabolites in red marks are only related to drug stress; metabolites in blue marks are only related to strain type or drug resistance level; metabolites marked green are associated with the three described above.

Amino acids are key precursors for the synthesis of important low molecular weight nitrogenous compounds[13]. It has been reported that pyridoxal phosphate is not only able to regulated valine metabolism by combining with the pyridoxal protein, but also to activate VB6 as a catalyst[14, 15]. VB6, as a singlet oxygen scavenger, can protect fungi from the oxidative damage, and it has been considered as a potential antioxidant[16]. Therefore, the observed changes of valine may be associated with a stimulation of the activity of VB6. Previous studies have reported that proline, being a kind of universal antioxidant, could protect filamentous fungi and yeast against oxidative stress[17, 18]. Proline is also able to reduce the production of reactive oxygen species in the mitochondria[19, 20]. In our study, on the one hand, the down-regulation of proline in the R group may suggest the idea that resistant strains had a wider oxidative stress tolerance than sensitive strains. On the other hand, proline was found at higher levels both in resistant and sensitive strains after tretment with ketoconazole. We suspected that proline is induced for protective purposes under stress conditions, such as drug stimulation. The redox system plays an important role in the occurrence and progress of several diseases, and glutathione is an indispensable compound that maintains redox homeostasis responding to oxidative stress[21]. In our study, glutathione was significant up-regulation in resistant strains after ketoconazole intervention. We speculated that such alternations might be associated with endogenous reactive oxygen species (ROS) production. Resistant strains synthesized a large amount of glutathione in response to endogenous ROS production in cells, thus reducing the sensitivity of antifungal agents. It is well known that yeasts activate a series of protective defense measures in response to external environment stimulations, such as the rapid accumulation of polyols (e.g. glycerin, arabitol, sorbitol and mannitol). For example, *C*. *albicans* secrets a large amount of glycerin and arabitol in response to osmotic stress, temperature and oxidative stress[22, 23]. Therefore, the up-regulation of glycerin and arabitol both in resistant and sensitive strains after tretment with ketoconazole suggested that *C*. *albicans* cells againsted the oxidative stress through the accumulation of polyols (Fig 7).

Sphingosine, phytosphingosine and sphinganine were observed up-regulation only in sensitive strains after KCZ intervention (Fig 7). Sushma *et al*. have reported that the deletion of the sphingolipid biosynthetic pathway genes FEN1 and SUR4 of *C*. *albicans* resulted in a major sensitivity to amphotericin B (AmB) than parent strains[24]. Vinay K. *et al*. have demonstrated that the modulation of AmB resistance by PMP3 was dependent on sphingolipid biosynthetic pathway, since AmB sensitivity of PMP3 deletants was suppressed by phytosphingosine, a sphingolipid pathway intermediate[25]. As a signal molecule, phytosphingosine-1-P could enhance the miconazole drug efflux to reduce the sensitivity. Several studies have showed that the overexpression of drug transporters is one of the main resistant mechanisms for several pathogens[26, 27]. As a consequence, we may speculate that the up-regulation of phytosphingosine-1-P in *C*. *albicans* can reduce its sensitivity to antifungal drugs by increasing the drug efflux. These results all indicated that sphingolipid biosynthetic pathway is essential for the achievement of drug resistance in *C*. *albicans*, highlighting that cell membrane is a main target for antibacterial action in *C*. *albicans*.

Furthermore, phospholipid metabolomics showed that resistant strains of *C*. *albicans* had high content of PC, PE, PG and low content of LysoPC, LysoPE, LysoPG. These results imply that phospholipase B is strongly expressed and the resistant strains probably enhanced its pathogenicity by this high expression[28]. Intracellular drug content is strictly related with the expression level of membrane transporters, and the rate of transported drugs depends on the fluidity of cell membrane[29]. This parameter is linked to the length of the lipid C-chain: the longer is the C-chain, the weaker is the fluidity of the cell membrane. We found that resistant strains of *C*. *albicans* had the highest content of glyceryl phosphatide fatty acids with more than 34-C. This content was significantly reduced after the treatment with ketoconazole in both resistant and sensitive groups. This suggests that strains can reduce the drug uptake by enhancing the cell membrane fluidity. At the same time, we also observed that phosphoglycerides containing an odd number of C atoms were down-regulated in resistant strains group. This is in good agreement with a previous study showing that phospholipid fatty acid with an odd number of C atoms affected the sensitivity of *C*. *albicans* against drugs[30].

Mitochondria play an important role in multiple processes, such as hyphal development, resistance to stress, virulence, apoptosis and sensitivity of yeast cells to drugs[31–33]. Cardiolipin and its precursor PG, the principal anionic phospholipid in mitochondrial membranes, are key elements in the response conferring resistance to osmotic and thermal stresses[34, 35]. We found that PG was increased in treatment groups, thus maybe contributing to mitochondrial defects, which probably reduce the content of antifungal drugs through the regulation of the expression of transporters[36]. Shingu-Vazquez *et al*. have found that mitochondrial dysfunction in some fungi, such as *S*. *cerevisiae* and *C*. *glabrata*, could lead to drug resistance, especially in reducing susceptibility to azole and polyene drugs[31]. Phosphatidylcholine is a precursor of lysophosphatidylcholine, which beyond the control of mitochondrial functions regulates intracellular vesicle trafficking[37, 38]. LysoPC was demonstrated to be able to inhibit the transition of *C*. *albicans* from yeast to hyphae *via* the MAP kinase pathway, but did not affect the growth of either yeast or hyphae[39]. Compared to the sensitive strains, we observed that lysoPC was up-regulated in the resistant group, thus indicating that lysoPC may be associated with *C*. *albicans* resistance.

## Conclusions

In this study, different MS-based approaches were used to investigate the metabolic profile associated with azole resistance in *C*. *albicans*. Our results allowed, as a model containing potential biomarkers changing in azole resistance in *C*. *albicans*. that the majority of these potential biomarkers were involved in metabolic processes related to amino acid metabolism, sphingolipid metabolism and phospholipid metabolism. It is a complex process probably involving, reducing the endogenous ROS production, strengthening the expression of drug transporters, changing the function of cell membranes and mitochondria. Overall, our study explores global changes in metabolites involved in resistance to azole drugs, providing a more detailed understanding of the evolution of drug resistance in *C*. *albicans*.

## Supporting information

S1 Table The raw data of extracellular metabolites from UHPLC-Q-TOFMS. (XLS)

S2 Table The raw data of intracellular metabolites from UHPLC-Q-TOFMS. (XLS)

S3 Table The raw data of intracellular metabolites from GC-MS. (XLS)

S4 Table The raw data of intracellular metabolites from HILIC-QQQ/MS. (XLS)
